# Eukaryotic Ribosome Biogenesis: The 60S Subunit

**DOI:** 10.32607/actanaturae.11541

**Published:** 2022

**Authors:** A. A. Moraleva, A. S. Deryabin, Yu. P. Rubtsov, M. P. Rubtsova, O. A. Dontsova

**Affiliations:** Shemyakin-Ovchinnikov Institute of Bioorganic Chemistry of the Russian Academy of Sciences, Moscow, 117997 Russia; Lomonosov Moscow State University, Faculty of Chemistry, Moscow, 119991 Russia; Skolkovo Institute of Science and Technology, Moscow, 121205 Russia

**Keywords:** nucleolus, ribosome, biogenesis, ribosomopathy

## Abstract

Ribosome biogenesis is consecutive coordinated maturation of ribosomal precursors in the nucleolus, nucleoplasm, and cytoplasm. The formation of mature ribosomal subunits involves hundreds of ribosomal biogenesis factors that ensure ribosomal RNA processing, tertiary structure, and interaction with ribosomal proteins. Although the main features and stages of ribosome biogenesis are conservative among different groups of eukaryotes, this process in human cells has become more complicated due to the larger size of the ribosomes and pre-ribosomes and intricate regulatory pathways affecting their assembly and function. Many of the factors involved in the biogenesis of human ribosomes have been identified using genome-wide screening based on RNA interference. A previous part of this review summarized recent data on the processing of the primary rRNA transcript and compared the maturation of the small 40S subunit in yeast and human cells. This part of the review focuses on the biogenesis of the large 60S subunit of eukaryotic ribosomes.

## INTRODUCTION

The first part of this review describes in detail the mechanisms of formation and processing of the common 90S precursor, biogenesis of the small 40S subunit, and the nucleolus as a special intranuclear structure necessary for the formation and early maturation of ribosome precursors. In this second part, we continue with a discussion of the details of ribosome biogenesis as exemplified by the formation of the large 60S ribosomal subunit in human and yeast cells.

## BIOGENESIS OF THE 60S SUBUNIT PRECURSOR

**Fig. 1:**
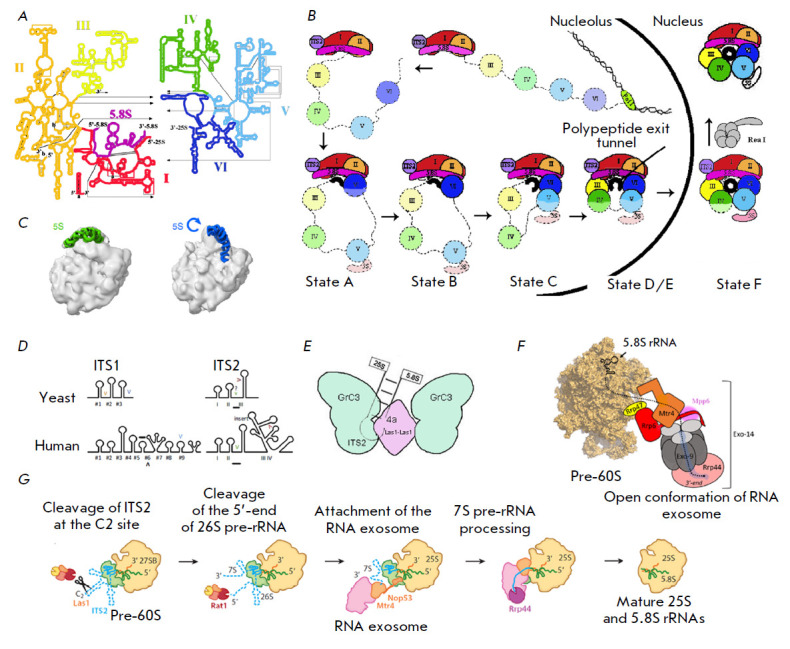
Structure and maturation of yeast pre-rRNA. (*A*) 25S rRNA contains six secondary structure domains (I–VI). 5.8S rRNA (shown in black) forms a complementary interaction with domain I of 25S rRNA (adapted from https://crw-site.chemistry.gatech.edu/). (*B*) Scheme of assembly of pre-60S pre-rRNA domains. The color coding of 25S rRNA domains is the same as in (*A*). Attachment of ribosomal proteins and biogenesis factors to the 35S rRNA precursor. The formation of the polypeptide exit tunnel (black circle) begins with binding of domain VI to domains I and II and a 5.8S region of the rRNA precursor. Folding of rRNA domains occurs in the following order: VI, V, III, and IV. In the F (final) state, domain V is completely folded [1]. (*C*) 5S rRNA turn [2]. (*D*) Secondary structures of yeast and human ITS1 and ITS2. Cleavage sites are marked with “V”. The predicted sites are indicated by question marks, and human exonuclease binding sites are underscored [3]. (*E*) Model of ITS2 processing by PNK RNase [4]. (*F*) Scheme of the interaction between the nuclear RNA exosome and pre-60S [5]. (*G*) Removal of ITS2 from the pre-60S particle by RNA processing enzymes. Intermediates formed during ITS2 removal are shown [6]

The 25S ribosomal RNA (rRNA) of the 60S yeast subunit consists of six conserved domains (I–VI) that are more closely intertwined than the 18S rRNA domains in the small subunit (SSU) (*Fig. 1*). Domains I and II of 25S and 5.8S rRNAs are located on the outer surface of the large subunit (LSU), and domains IV and V are involved in the functional centers. Domains III and IV connect the small and large subunits. In this case, rRNA domain III binds to other rRNA domains in the lower part of the 60S subunit, the 5.8S rRNA is located between domains I and III, and the 5S rRNA is anchored at the top of domains II and V (*Fig. 1*). Domain VI is connected to domains I and II and 5.8S rRNA.

**Fig. 2:**
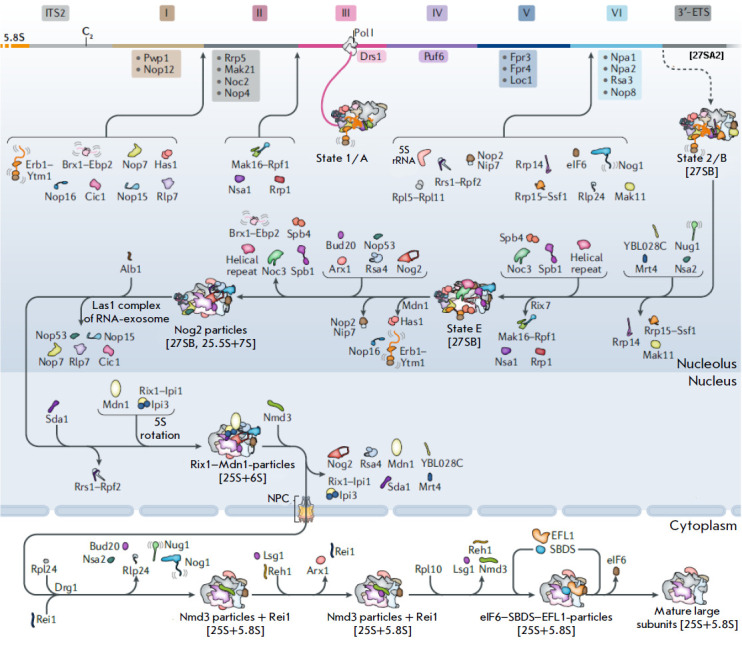
Large ribosomal subunit assembly in yeast. Consecutive stages of large ribosomal subunit (60S) maturation are shown, starting with the earliest stages in the nucleolus, through stages in the nucleoplasm, and finally in the cytoplasm. rDNA regions giving rise to 5.8S rRNA, ITS2, domains I–VI of 25S rRNA, and 3’-ETS are indicated. Adapted from [14]. Assembly factors and complexes with known structures are depicted as cartoons; those whose structures are not known are indicated with text only

**Fig. 3:**
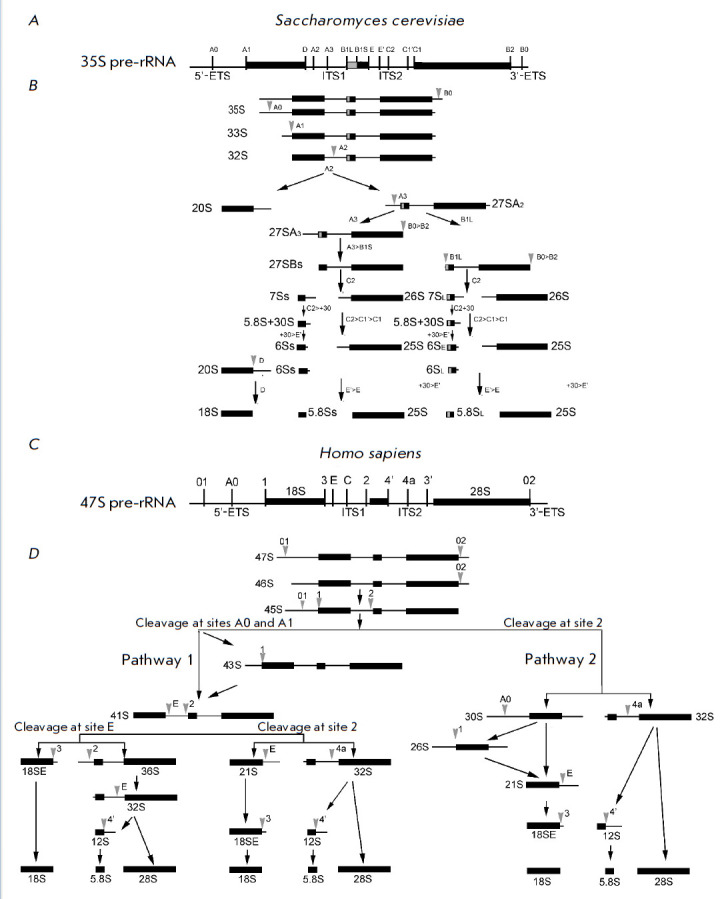
Maturation pathways of the 35S pre-rRNA transcript in *Saccharomyces cerevisiae *(*A*) and the 47S pre-RNA transcript in *Homo sapiens *(*C*). Three of the four rRNAs (18S, 5.8S, and 25S (in yeast)/28S (in humans)) are synthesized by Pol I as a single long transcript. The coding sequences of mature rRNAs are flanked by 5’- and 3’-ETS, ITS1, and ITS2 non-coding spacers. The schematic shows the relative position of known and predicted cleavage sites. (*B*) Processing of pre-rRNA in budding yeast. (*D*) A simplified schematic of human pre-rRNA processing. The primary transcript, 47S pre-rRNA, is initially cleaved at both ends at sites 01 and 02 to form the 45S precursor that is processed via two alternative pathways [51]. “>” (e.g., C2>C1’>C1) denotes consecutive shortening of the appropriate 3’- or 5’-ends of the pre-rRNA by nucleases

In 2017, three research groups published high-resolution cryo-EM structures of pre-60S from yeast nuclei. Six types of pre-60S particles have been identified in these structures. They differ in the packing density of RNA and the composition of ribosomal proteins (RPs) [1, 6, 7, 8] (*Fig. 1*). The secondary structure of LSU rRNA comprises six domains; however, these domains cannot be clearly distinguished in the 3D structure, in contrast to the four domains of the SSU 18S rRNA. During transcription, domains I and II of the 25S rRNA bind 5.8S and ITS2 to form a structural scaffold for further assembly (*Fig. 1*) [1, 7, 8]. Immediately after transcription by RNA polymerase I (Pol I), domain VI folds into an ordered structure, while the central domains (III, IV, and V) remain disordered, interacting with the ribosome assembly factors (RAFs) that prevent contacts with 5’-terminal domains. In mature LSU, domains I–V form the peptide exit tunnel, domains II and VI form the GTPase center, and domain V forms the peptidyl transferase center (PTC) comprising the A and P sites. Coordinated binding and dissociation of various RAFs ensure a consecutive formation of these key structures. For example, a series of consecutive interactions with RAFs (Nog1, Rei1, and Reh1), which occur immediately after the formation of the polypeptide exit tunnel, promote the completion of folding [9 , 10, 11, 12, 13]. Domain VI, which corresponds to the 3’-end of 25S rRNA, is stably incorporated into the particle, closing the rRNA ring and leaving domains III–V free [1, 7, 8] (*Fig. 2*). They are consecutively assembled around the polypeptide exit tunnel, leaving the PTC in an immature conformation. This sequence of events differs from 40S biogenesis, where rRNA folding occurs consecutively from the 5’- to the 3’- end of 18S rRNA. Notably, the essential condition for the formation of these ring rRNA intermediates in the 60S subunit is the removal of the internal transcribed spacer 1 (ITS1) and external transcribed spacer (3’-ETS) (*Fig. 3*), because these sequences sterically prevent the association of rRNA domain VI with other domains. The ring intermediate comprises both the 5’- and 3’-ends of rRNA and can protect rRNA from degradation but does not interfere with the modification of heterocyclic bases. Anchoring of the 5’- and 3’-ends probably facilitates the assembly of mobile neighboring domains, forming a kind of scaffold. Domain V especially benefits from the preassembly of other rRNA domains, because its regions should form contacts with several domains, including 5S rRNA (*Fig. 1, Fig. 2*). During this process, the conformation of this complex changes three times (*Fig. 1, Fig. 2*).

Some RAFs, such as Rrp5, Mak21, Noc2, and Nop4, seem to promote rRNA compaction at the earliest co-transcriptional stages of LSU biogenesis, forming a rigid support for coordinated RNA folding [14, 15, 16, 17, 18, 19]. The structures of pre-ribosomal particles in mutants deficient in these RAFs have a looser structure [14, 18]. Early RAFs (Npa1, Npa2, Rsa3, and Nop8) and RNA helicase Dbp6 form a stable complex capable of performing a structural function [19, 20]. Six other RNA helicases (Dbp2, Dbp3, Dbp7, Dbp9, Mak5, and Prp43) are also required at the initial assembly stages that involve the remodeling of RNA structures (for review, see [20, 21]). Interestingly, cleavage of ITS1 at A2 and A3 is associated with the transcription and processing of sequences that are separated from each other by several thousand nucleotides in the primary structure. Co-transcriptional cleavage at site A2 occurs after synthesis of 25S rRNA domains I and II [22, 23]. Hydrolysis at A3 occurs after the completion of 3’-ETS transcription and processing [24]. Probably, protein-mediated RNA folding results in the formation of structures that can interact with RAFs and nucleases. For example, Rrp5 binding to ITS1 both in the SSU processome (site A2) and in pre-60S particles (site A3) [25, 26, 27] can regulate cleavage at these sites and coordinate the assembly of both subunits [16, 18, 28, 29].

Early nucleolar pre-60S particles contain approximately 30 RAFs and 30 ribosomal proteins (*Table*). Most of them seem to stabilize the structure, and some exhibit enzymatic activity that controls the transition between key steps in the 60S assembly process. For example, the Nop2 and Spb1 factors are important for snoRNP-independent RNA methylation. The substrate and function of helicase Has1 have not been identified. The functions of GTPases Nog1 and Nug1, which are likely required for the release of Nop2 and Spb1 from later pre-60S subunits, have not been identified. Interestingly, Brix family proteins and their partner proteins [31, 32, 33, 34] probably fold rRNA by bringing different domains together. For example, the Ssf1–Rrp15 dimer binds rRNA domains III and VI; the Brx1–Ebp2 complex binds the junction of domains I and II; Rpf1–Mak16 comes into contact with 5.8S rRNA and domains I, II, and VI. Brix family proteins, Rpf2 and Rrs1, interact with 5S rRNA and domain V in the pre-60S Nog2 particle [13], and the Imp4–Mpp10 complex binds 5’-ETS and the nascent 3’-domain in the 90S particle.

**Table:** Large ribosomal subunit assembly factors [20, 30]

Ribosome biogenesis factors; LSU components in Saccharomyces cerevisiae					
Cluster number			Homo sapiens	S. cerevisiae	Function
8	8	4	PDCD11	Rrp5	Structural
	4		RBM28	Nop4	Structural
1			DDX51	Dbp6	DEAD-box-helicase
	1		DDX50	Dbp3	«
1	1		DDX31	Dbp7	«
1	4		DDX56	Dbp9	«
1	1		DDX24	Mak5	«
			DDX54	Dbp10	«
	2		GAR1	Gar1	Pseudouridine synthase cofactor
2	2		NHP2	Nhp2	Pseudouridine synthase cofactor
	8		NOP10	Nop10	Pseudouridine synthase cofactor
6	6	6	DKC1	Cbf5	Pseudouridine synthase
2	2	2	NOP56	Nop56	Main component of C/D Box snoRNP
			NOP58	Nop58	Same
2	2	2	FBL	Nop1	«
2	2	11	NHP2L1	Snu13	«
			KIAA0020	Puf6	Structural
1			PWP1	Pwp1	Structural
			RBM34	Nop12	Structural
4	4	4	DDX27	Drs1	DEAD-box-helicase
6	11	11	PAK1IP1	Mak11	Structural
			PPAN	Ssf1	«
			PPAN	Ssf2	«
4	4	4	RRP15	Rrp15	«
9	11		SURF6	Rrp14	«
4	4	4	WDR74	Nsa1	«
4	4	4	RRP1/NOP52	Rrp1	«
4	10	10	RPF1	Rpf1	«
4	4	4	MAK16	Mak16	«
			NVL	Rix7	AAA-ATPase
4	4	4	EBNA1BP2	Ebp2	Structural
4	4	4	BRIX1	Brx1	«
4	4	4	BOP1	Erb1	«
		4	WDR12	Ytm1	«
8	8	8	DDX18	Has1	DEAD-box-helicase
4	4	11	NOC2L	Noc2	Structural
1			FTSJ3	Spb1	rRNA methyltransferase
			DDX55	Spb4	DEAD-box-helicase
1			NOP2	Nop2	rRNA methyltransferase
1			NIP7	Nip7	Structural
			NOC3L	Noc3	«
4	4	4	PES1	Nop7	«
4	4	4	MKI67IP	Nop15	«
				Cic1	«
	8		eIF6	eIF6	«
11	11	11	GLTSCR2	Nop53	Structural, binding of RNA-exosome
2			RSL24D1	Rlp24	Structural
4	4	4	GTPBP4	Nog1	GTPase
			MRTO4	Mrt4	Structural
4	1	1	NSA2	Nsa2	Structural
1			GNL3	Nug1	GTPase
11		11	RRS1	Rrs1	Structural
		1	RPF2	Rpf2	Structural
11		11	GNL2	Nog2	GTPase
			NLE1	Rsa4	Structural
			WDR18	Ipi3	Structural
			MDN1	Mdn1	AAA-ATPase
11	11		SDAD1	Sda1	Structural
			Nmd3-containing particles		
2			NMD3	Nmd3	«
		2	ZNF622	Rei1	«
			ZNF622	Reh1	«
	6		LSG1	Lsg1	ATPase

Isolation of the Nsa1–pre-60S complex revealed that, during LSU formation, the Nsa1–Rpf1–Mak16– Rrp1 complex stabilizes the surface exposed to the solvent; the Rlp24–Nog1–Mrt4–Mak16–Tif6–Nsa2 complex interacts predominantly with domains V and VI; and the Nsa3–Nop15–Rlp3–Nop7–Erb1–Ytm1 complex organizes ITS2 during foot formation. Like several RAFs of the 90S subunit, Erb1 has a long N-terminus that meanders over the pre-60S surface, contacting distant factors, including the Brx1–Ebp2 dimer, Has1 helicase, Nop16, and foot factor Nop7 [1, 7, 8]. Furthermore, the β-propeller domain of Erb1 interacts stably with the Ytm1 factor that is a substrate for the Rea1 ATPase [35]. At a certain stage, Rea1 creates a mechanochemical force to remove Ytm1 and the deep-rooted Erb1. Notably, other protein complexes also contain proteins (Nsa1, Rlp24) dissociation of which requires AAA-ATPases such as Rix7 and Drg1 [35, 36].

It is not yet clear when and how the 5S RNP (5S rRNA, uL18/Rpl5, uL5/Rpl11) is incorporated into the earliest pre-60S particles. The interaction occurs with the 5S RNP in a folded conformation, and, therefore, it requires a 180° conformational rotation at later stages of 60S maturation [6, 13, 37]. This stage combines with the formation of PTC correct occurrence, which is checked through removal of Rsa4 by the huge Rea1 AAA-ATPase and GTP-dependent dissociation of Nug2 [38, 39]. Binding of nuclear export factors to pre-60S and subsequent transport occur after passing the assembly quality control stages [39]. Despite a strict system for assembly accuracy control in the nucleus, pre-60S particles containing ITS2 and related factors can enter the cytoplasm and even participate in translation [40,41, 42].

**Transport of pre-60S into the cytoplasm and quality control of subunit precursors**

Transport from the nucleolus to the nucleoplasm is accompanied by the exchange of protein factors that promote remodeling and subsequent export of precursors from the nucleus. In the cytoplasm, pre-60S ribosomes undergo the final stages of maturation; in particular, removal of RAFs, attachment of the last few RPs, and quality control of the functional centers.

The nuclear export adaptor protein Nmd3 controls the interaction between Crm1/Xpo1 exportin and the 60S subunit, which facilitates transport of the subunit into the cytoplasm [6, 43, 44, 45, 46]. The interaction between 60S subunits and noncanonical export factors has been reported [6, 46].

In the cytoplasm, the pre-40S precursor binds to several RAFs, which block access to the mRNA channel and P-site for initiator tRNA binding, and undergoes quality control. Subsequently, 40S binds to the 60S large subunit using the Fab7 ATPase and eukaryotic translation initiation factor 5B (eIF5B). In this case, the GTPase center of eIF5B should be in an active conformation. The formation of the complex ensures the ability of mature 40S to hydrolyze GTP. The formation of the mature 3’-end of 18S rRNA by endonuclease Nob1 is accompanied by a dissociation of the remaining RAFs from 40S and dissociation of the 40S–60S complex, which is an indication that the small subunit is ready for the final stage of processing [12, 47, 48, 49].

**Human ribosome biogenesis is far more complex than yeast ribosome biogenesis**

The main stages and molecular events of ribosome biogenesis are conserved. For a long time, it was believed that most stages of subunit formation in human and *Saccharomyces cerevisiae *cells are identical, but this turned out to be an oversimplification of the situation. Human nucleoli have three compartments, instead of two in yeast’s nucleoli, are involved in a greater number of cellular processes [50, 51], and contain at least 20-fold more proteins than yeast (up to 300 in yeast; 6,000 in humans) [52]. The complexity of the physiological processes in multicellular organisms determines the need for new modes for regulating ribosome formation, which is evidenced in particular by the dependence of 40S subunit synthesis on circadian rhythms in mice [53, 54].

Human ribosomes are larger than yeast ribosomes. They contain more ribosomal proteins that are often larger than yeast proteins. Human rRNAs are comparable in size to yeast rRNAs, except for the 28S rRNA that is 1.5-fold larger. ETS and ITS sizes differ most significantly: in humans, they contain many monoand dinucleotide repeats that may have arisen due to replication errors. The more complex ribosomal structure in higher eukaryotes and, accordingly, the rRNA structure inevitably affect ribosome biogenesis [26], which is reflected in a larger number of precursors [55]. Biogenesis of human 40S subunits is accompanied by the formation of at least two additional precursors containing 30S and 21S pre-rRNAs (*Fig. 3*) [15, 56]. In yeast, 70–80% of nascent pre-rRNA transcripts undergo co-transcriptional cleavage in ITS1, while the primary transcript in mammals is usually cleaved post-transcriptionally [23, 57]. ITS1 processing in human cells has been shown to be more complex than that in yeast cells and require both endo- and exonucleolytic activity [57, 58 , 59].

A distinctive feature of eukaryotic ribosome biogenesis is the modular assembly of pre-ribosomal complexes. Both in yeast and in humans, the UTP-A, UTP-B, UTP-C, U3 snoRNA, RCL1–BMS1 heterodimers, and IMP3–IMP4–MPP10 and EMG1 complexes are assembled on the newly synthesized pre-rRNA transcript and form the core of the so-called SSU processome. Some complexes, such as human PeBoW (Nop7–Erb1–Ytm1 in yeast) [60] and PELP1– TEX10–WDR18 (Rix1–Ipi3–Ipi1 in yeast) [61], act similarly during the biogenesis of pre-60S subunits. Despite their evolutionary conservatism, their composition is different in various species; in humans, several additional RNA helicases, e.g., DDX21 for UTP-B and DDX27 for PeBoW, have been identified [62, 63]. All these facts indicate additional remodeling steps at the early stages of pre-ribosome assembly in humans.

Production of 18S rRNA in mammalian cells can occur upon suppression of 28S rRNA synthesis [64, 65, 66, 67]. Depletion of several human LSU ribosomal proteins [57] does not prevent the formation of both 18S rRNA and its direct precursor, 18S-E pre-rRNA, despite a serious decrease in 28S rRNA synthesis. These data support a model in which early assembly events in each ribosomal subunit control proximal cleavage in ITS1. Notably, this mode of splitting the SSU and LSU precursors does not preclude the existence of factors that may be involved in both ITS1 cleavages. In mammalian cells, separation of the SSU and LSU precursors occurs simultaneously, which complicates the analysis of processing stages. Depletion of various mouse SSU and LSU assembly factors leads to the inhibition of one of the two ITS1 cleavages [68]. It is hypothesized that cleavage in mouse pre-rRNA at two ITS1 sites, which correspond to human E and C sites, is coordinated with early assembly in the SSU or LSU. As a result, each subunit remains attached to ITS1 until it reaches the maturation stage and is capable of cleaving the ITS1 [68].

In the absence of several assembly factors, the LSU inhibits cleavage at the A2 site, which leads to the accumulation of aberrant 35S pre-rRNAs [69, 70, 71] and processing arrest. In contrast to yeast, transcript cleavage in mammalian cells occurs at either of two ITS1 sites, which leads to the generation of major precursors that mature to 18S and 5.8S/28S rRNAs (*Fig. 3*). Defects in the early steps of LSU assembly in mammalian cells inhibit cleavage in the 3’-region of ITS1. Separation of RNA ribosomal subunits in mammals involves cleavage of ITS1 at two sites, as opposed to one in yeast.

Quite little is known about the structure of human pre-ribosomes, because there are no reliable methods for their isolation and purification. Many human ribosome synthesis factors have been identified using high-throughput small interfering RNA screening capable of detecting defects in the production of pre-rRNA intermediates and accumulation of ribosomes or pre-ribosome components in the nucleolus or nucleoplasm [30, 72]. Such screening has identified 286 proteins, including yeast RAF orthologues, as well as 74 human-specific proteins and snoRNAs which may be RAFs [30, 73] (*Table*). Recently, 139 potential RAFs have been identified by screening for factors that affect the amount or morphology of nucleoli [74]. However, the role of individual human RAFs has barely been studied. The composition, activity, and structure of intermediate complexes are also not well understood, because most data have been obtained by extrapolating data from the analysis of yeast pre-ribosomes. In some cases, the functions of even homologous ribosome synthesis factors may differ; for example, yeast Nip7 and Spb1 are required for the maturation of 5.8S and 25S rRNAs, and their homologues, human NIP7 and FTSJ3, are involved in the synthesis of 18S rRNA [75]. A separate issue is the difficulty in identification of RAFs directly involved in subunit assembly and how they differ from the proteins/signaling pathways that indirectly affect the production of ribosomes.

A high-throughput screening of the functions of human nucleolar proteins was performed by reducing their level using small interfering RNAs. According to the results of such screening, nucleolar proteins may be divided into 12 functional clusters, depending on their influence on certain stages of pre-rRNA processing. Similar defects were observed in different cell types, including primary cell lines [30]. For example, UTP18-depleted cells accumulate aberrant 34S pre-rRNA due to the inhibition of early cleavages of the rRNA precursor (at sites 01, A0, and 1). RPS11- depleted cells accumulate significant amounts of 30S pre-rRNA due to the lack of processing at sites A0 and 1. NOL9 is primarily involved in ITS2 processing, because 32S pre-rRNA accumulates in its absence. 43S and 26S pre-rRNAs are present in higher amounts in RPS3-depleted cells than in control cells, which indicates that this protein is involved in the cleavage at the A0 and A1 sites. RPS3-depleted cells accumulate a truncated 21S-21S-C form (*Fig. 3*).

The human MDN1, NVL2, and AFGH2 proteins are homologues of the three yeast AAA-ATPases (Rea1/ Mdn1, Rix7, and Drg1, respectively) involved in the release of specific biogenesis factors from pre-60S particles [76]. The presence of MDN1 in the pre-60S and PELP1–TEX10–WDR18 complexes (Rix1 complex in yeast) suggests that this enzyme acts similarly in different species, from yeast to humans [77]. Some RNA helicases also play common roles. For example, yeast Dhr1 and human DHX37 mediate the release of U3 snoRNA [78, 79, 80, 81]. In this case, several human RNA helicases have additional functions associated with ribosome biogenesis. For example, DDX51 is required for the release of U8 snoRNA, which is specific to multicellular organisms, from pre-LSU complexes [82], while DDX21 coordinates pre-rRNA processing with transcription, facilitating access of late snoRNA pre- 40S to the complexes [63, 78, 83].

Several new pre-ribosomal mini-complexes have been identified in human cells [82]. For example, the anti-apoptotic transcription factor AATF, neurohydin (NGDN), and NOL10 form a nucleolar subcomplex (ANN) [84]. These proteins interact with early pre-ribosomes, and the lack of any of the ANN components leads to impaired pre-rRNA cleavage in the early stages of biogenesis. XND, a nucleolar complex consisting of the NF-kB repressing factor (NKRF), RNA helicase DHX15, and 5’–3’-exonuclease XRN2, is also involved in the early stages of human ribosome assembly [85]. NKRF recruits XRN2 to pre-ribosomal complexes, where it is involved in pre-rRNA processing and removal of excised pre-rRNA fragments. NKRF also stimulates the ATPase and helicase activities of DHX15 [85]; i.e., these proteins seem to function in tandem in the early stage of pre-rRNA remodeling. A yeast homologue of DHX15, Prp43, is involved in snoRNA release from pre-60S particles and promotes cleavage of the 3’-end of 18S rRNA [86, 87]. The NF45–NF90 heterodimer, a transcription factor, binds double-stranded RNA within pre-60S. The lack of these factors does not affect rRNA processing, but it causes nucleolar morphology changes and accumulation of pre-60S complexes [88].

Recently, cryo-EM structures of late nuclear and cytoplasmic complexes of the human pre-40S subunits were obtained in the Beckman laboratory [89]. The structure of one of the intermediate states revealed the position of the biogenesis factor RRP12 and two methyltransferases (BUD23 and TRM112) in the head of the 40S subunit. The later human cytoplasmic pre-40S particle is very similar to yeast pre- 40S, with conserved RAFs in identical positions. Thus, the pre-40S structure and the final 18S rRNA processing mechanism are evolutionarily conserved [89].

**Ribosomal proteins and their role in the formation of the rRNA structure and subunit maturation**

The main role of ribosomal proteins is to maintain the structure and function of ribosomes and the production of active ribosomes. Mathematical modeling has shown a great advantage in assembling elaborate complexes – in particular ribosomes – from numerous small ribosomal proteins, rather than bundling a small number of larger polypeptides [90]. Most human RPs are known to have a single variant, while many yeast RPs have two isoforms. Surprisingly, ~50% of the transcripts synthesized by human RNA polymerase II are RP mRNAs [91] and concentrations of 80 RPs in the cell are carefully maintained at levels optimal for ribosome assembly. Most RP genes comprise one or more common promoter elements (GABP, Sp1, YY1) to synchronize transcription. The mRNAs of all RPs contain a 5’-terminal oligopyrimidine tract (5’-TOP), which also enables co-regulation of their translation [92]. Ribosomal proteins are usually positively charged and prone to aggregation and degradation. Chaperones bind (often co-translationally) to newly synthesized RPs, stabilize them, and facilitate import into the nucleus and attachment to pre-ribosomal complexes [93, 94]. Homologues of many yeast RP chaperones have been found in human cells: the Bcp1/BCCIP, Syo1/HEATR3, Rrb1/GRWD1, Sqt1/ AMMP, and Tsr2/TSR2 proteins. However, others, such as Acl4 and Yar1, apparently were not preserved in multicellular organisms [78, 93, 95, 96, 97, 98]. Notably, the ribosomal proteins RPL5 (uL18) and RPL11 (uL5) bind to pre-ribosomes as a subcomplex together with 5S rRNA [99]. Pre-5S rRNA is synthesized by RNA polymerase III, and maturation of its 3’-end requires the REX1, REX2, and REX3 exonucleases, as well as RPL5 [100, 101, 102]. In both yeast and humans, Rrs1/ RRS1 and Rpf2/BXDC1 are required for 5S RNP integration into pre-60S complexes and the tumor suppressor protein PICT1/GLTSCR2 is an additional factor in human cells [102, 103]. The interaction of many RPs with pre-ribosomes is initially unstable, but the correct folding and formation of tertiary structures in rRNAs gradually lead to their stable incorporation into ribosomal complexes. A distinctive feature of ribosome assembly, which is preserved not only in eukaryotes, but also occurs during the synthesis of prokaryotic ribosomes [104], is the hierarchical incorporation of RPs, which promotes the sequential organization of individual subunit domains. First, proteins of the 5’-, central, and 3’-minor domains of 18S rRNA form the SSU body, and then the head and beak are assembled [105]. Similarly, RPs located on the LSU surface exposed to the solvent are incorporated in the structure at the first stages of assembly, while the proteins that bind to the intersubunit interface and central prominence are incorporated later [106]. The universal nature of the hierarchical incorporation of RPs suggests that the stepwise assembly, stabilization, and compaction of various ribosomal subunit domains are an important mechanism that ensures correct progression along the assembly pathway.

## CONCLUSIONS AND OUTLOOK

For many years, the complex biogenesis pathway of the eukaryotic ribosome had been studied mostly in yeast cells, where the simplicity of genetic manipulations and the possibility of isolating large amounts of pre-ribosomal complexes for compositional and structural analysis provided a wealth of data on the fundamentals of ribosome assembly. Recent studies have confirmed that many stages of ribosome assembly in yeast and humans are important information about the specific biogenesis stages that have undergone adaptation during evolution. Although many of the factors necessary for human ribosome biogenesis have been identified, it is likely that the list of RAFs will significantly expand. The main challenge is to determine which of the factors necessary for ribosome synthesis are directly associated with pre-ribosomal complexes and to analyze the individual roles of such proteins during subunit assembly. Recent cryo-EM structures of yeast pre-ribosomes have provided a wealth of information on the temporal order, distribution, and molecular functions of many RAFs. Structural analyses of pre-ribosomes should significantly improve our understanding of human ribosome assembly.
